# Manipulating the air-filled zebrafish swim bladder as a neutrophilic inflammation model for acute lung injury

**DOI:** 10.1038/cddis.2016.365

**Published:** 2016-11-10

**Authors:** Yuefei Zhang, Hongcui Liu, Junlin Yao, Yanfeng Huang, Shenlu Qin, Zheng Sun, Yingchun Xu, Shu Wan, Hongqiang Cheng, Chunqi Li, Xue Zhang, Yuehai Ke

**Affiliations:** 1 Research Center of Molecular Medicine, Department of Pathology and Pathophysiology, Program in Molecular Cell Biology, Zhejiang University School of Medicine, Hangzhou 310058, China; 2 Collaborative Innovation Center for Diagnosis and Treatment of Infectious Diseases, Hangzhou 310003, China; 3 Hunter Biotechnology Corporation, Hangzhou 310053, China; 4 Department of Pulmonology, Children's Hospital, School of Medicine, Zhejiang University, Hangzhou 310000, China; 5 Department of Neurosurgery, The 1st Affiliated Hospital, School of Medicine, Zhejiang University, Hangzhou 310000, China

## Abstract

Acute lung injury (ALI) and its more severe form, acute respiratory distress syndrome (ARDS), are life-threatening diseases that are associated with high mortality rates due to treatment limitations. Neutrophils play key roles in the pathogenesis of ALI/ARDS by promoting the inflammation and injury of the alveolar microenvironment. To date, *in vivo* functional approaches have been limited by the inaccessibility to the alveolar sacs, which are located at the anatomical terminal of the respiratory duct in mammals. We are the first to characterize the swim bladder of the zebrafish larva, which is similar to the mammalian lung, as a real-time *in vivo* model for examining pulmonary neutrophil infiltration during ALI. We observed that the delivery of exogenous materials, including lipopolysaccharide (LPS), Poly IC and silica nanoparticles, by microinjection triggered significant time- and dose-dependent neutrophil recruitment into the swim bladder. Neutrophils infiltrated the LPS-injected swim bladder through the blood capillaries around the pneumatic duct or a site near the pronephric duct. An increase in the post-LPS inflammatory cytokine mRNA levels coincided with the *in vivo* neutrophil aggregation in the swim bladder. Microscopic examinations of the LPS-injected swim bladders further revealed *in situ* injuries, including epithelial distortion, endoplasmic reticulum swelling and mitochondrial injuries. Inhibitor screening assays with this model showed a reduction in neutrophil migration into the LPS-injected swim bladder in response to Shp2 inhibition. Moreover, the pharmacological suppression and targeted disruption of Shp2 in myeloid cells alleviated pulmonary inflammation in the LPS-induced ALI mouse model. Additionally, we used this model to assess pneumonia-induced neutrophil recruitment by microinjecting bronchoalveolar lavage fluid from patients into swim bladders; this injection enhanced neutrophil aggregation relative to the control. In conclusion, our findings highlight the swim bladder as a promising and powerful model for mechanistic and drug screening studies of alveolar injuries.

Acute lung injury (ALI) and its devastating clinical syndrome, acute respiratory distress syndrome (ARDS), are characterized by rapid respiratory failure, refractory arterial hypoxemia, pulmonary edema and bilateral infiltrates accompanied by pleural effusions.^1^ Despite multiple advances over several decades, the mortality rate for severe ALI/ARDS remains high, at 45%.^2^ Since its initial description in 1967 and the establishment of the Berlin Definition in 2011, the underlying pathomechanism behind the onset and progression of ALI/ARDS has not been fully explored.^1, 3^

Pulmonary alveoli, air-filled sacs at the terminal ends of the distal airways, have recently garnered attention as unique compartments that are vulnerable to various environmental pathogenic attacks.^4^ Exogenous materials, such as bacteria, viruses and air pollutants, can enter the alveolar microenvironment and trigger ALI/ARDS.^5, 6, 7^ In addition to their important host defense functions, neutrophils play key roles in promoting ALI/ARDS progression by infiltrating the pulmonary alveoli and releasing granule proteins or reactive oxygen species.^8^ ALI is alleviated in animal models when neutrophilic chemotaxis, migration, adhesion and transcellular diapedesis are impaired.^9, 10, 11^ Despite decades of research, the underlying mechanism behind alveolar neutrophil infiltration during ALI remains unclear. This can be attributed in part to a lack of *in vivo* animal models for observing the dynamics of alveolar neutrophil recruitment.^12^

The larval zebrafish (*Danio rerio*) is a widely accepted and used animal model because of its unique advantages in genetic manipulation, real-time imaging and relatively high-throughput screening for small-molecule drugs.^13^ Owing to the optical transparency of zebrafish larvae, microinjection has been widely applied in fundamental research using zebrafish as an animal model.^14^ Furthermore, a fully automated robotic system for microinjection of zebrafish embryos were invented.^15^ Based on computer vision and motion control, the microrobotic system performs injection at a high speed. In addition, with the help of automated image-based phenotypic analysis in zebrafish, this swim bladder platform is amenable to automation necessary for high-throughput drug screening.^16^ Zebrafish models have been utilized to investigate the pathogeneses of lung diseases, including idiopathic pulmonary fibrosis modeled by embryo xenotransplantation,^17^ the host–pathogen interactions that occur after bacterial infections^18^ and tailfin injury-induced neutrophilic inflammation.^19^ Nevertheless, these studies do not provide direct and definitive evidence for the feasibility and effectiveness of zebrafish as an *in vivo* model for lung diseases.

The zebrafish swim bladder, which serves as a variable buoyancy device, shares similarities with the lung's anatomical structure,^20^ morphological development^21^ and transcriptional patterns.^22^ As with terrestrial lungs, epithelial surfactants are detected in this gas-filled organ.^21, 23^ The zebrafish swim bladder has been developed to investigate human mucosal^24^ and fungal infections.^25^ Intriguingly, *in vitro* data on swim bladder elastogenesis have suggested the practicality of this organ as an *in vivo* injury-repair model for lung studies.^26^ However, there have been no reports on the use of the swim bladder as an *in vivo* model for lung diseases prior to our study.

Protein kinases and phosphatases play critical roles in the signal transduction that occurs during neutrophil migration.^27^ By inhibiting relevant protein kinases or phosphatases, including PI3K,^28^ Erk and Jnk,^29, 30^ pulmonary neutrophil infiltration is reduced in the ALI animal model. Therefore, inhibitory screening of protein kinases and phosphatases may provide potential therapeutic targets for ALI-associated neutrophilic infiltration.

In this study, we used the zebrafish swim bladder as an *in vivo* system to study the pathogenesis of neutrophilic infiltration during ALI. A significant rise in neutrophil aggregation, inflammation and swim bladder injuries was detected in the lipopolysaccharide (LPS)-induced swim bladder injury model. Our screen for potential therapeutic targets revealed Shp2 involvement in neutrophil infiltration into the swim bladder. Moreover, pulmonary inflammation in ALI mice was alleviated by Shp2 inhibition and knockout. Microinjections of bronchoalveolar lavage fluid (BALF) from pneumonia patients into the swim bladders increased neutrophil recruitment relative to the control. These results highlight the swim bladder as a promising *in vivo* model for assessing the severities of lung diseases. Furthermore, a better understanding of pulmonary neutrophilic infiltration during ALI has been achieved using this model.

## Results

### Exogenous material injections trigger neutrophil migration into the swim bladder

Multiple ALI pathogenesis studies have described the use of several chemicals in mice to mimic specific stimuli. These include LPS, a well-established component of certain bacterial infections; Poly IC, a suitable mimic of viral infections; and silica nanoparticles (Nano-SiO_2_), a common mimic for air pollution.^31, 32^ Neutrophilic infiltration, one of the most threatening pathologic characteristics in ALI, indicates the acute early-stage inflammatory response.^33^ We traced neutrophil migration using a previously established *Tg(mpo:GFP)* zebrafish line that expressed the green fluorescent protein (GFP) under the control of the neutrophil-specific myeloperoxidase promoter.^34^ To model the aforementioned stimulation in zebrafish, we microinjected the exogenous materials into the swim bladders of 5-dpf (days post fertilization) larvae as previously described^35^ and investigated the neutrophil response.

**Figure 1:**
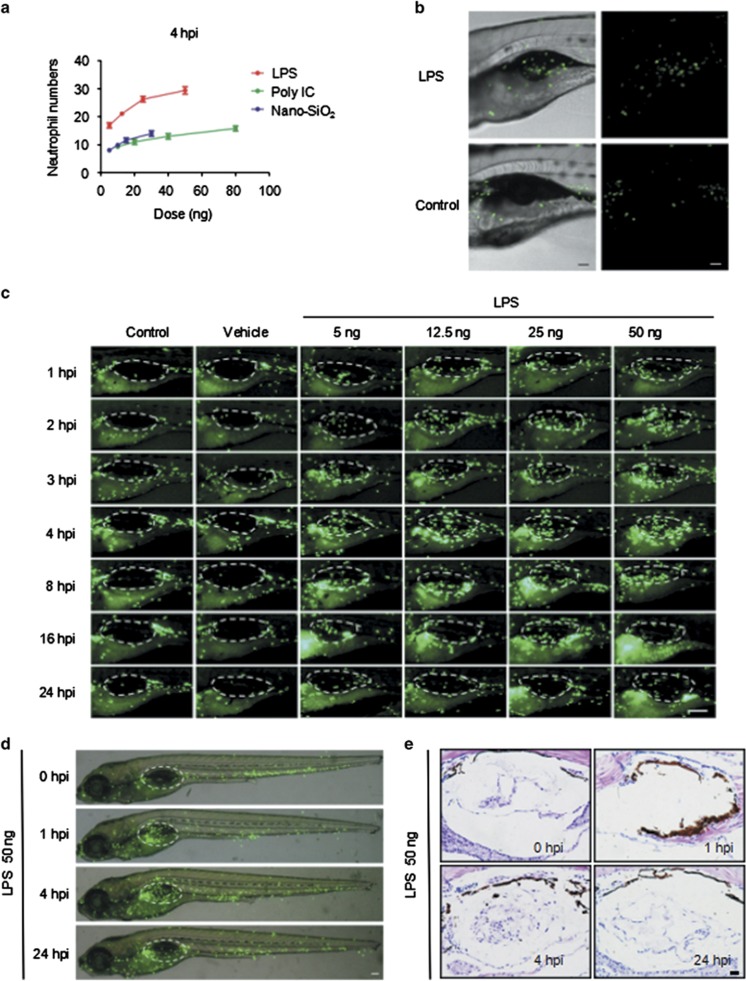
Neutrophils migrate into the LPS-injected zebrafish swim bladder. (**a**) The dose response for neutrophil recruitment into the swim bladder of 5-dpf zebrafish larvae after stimulation with LPS, Poly IC and Nano-SiO_2_ for 4 hpi. (**b**) Neutrophil accumulation into the *Tg(mpo:GFP)* zebrafish (5 dpf) swim bladder after the 50 ng LPS injection was imaged by confocal microscopy at 5 hpi (maximum projection slices *n*=66; green, neutrophil; scale bar: 60 *μ*m). (**c**) Neutrophil recruitment to the LPS-injected swim bladder (indicated by the dotted line) of *Tg(mpo:GFP)* zebrafish larvae at 5 dpf was monitored (green, neutrophil; scale bar: 100 *μ*m). (**d**) The neutrophil distribution (green) throughout the whole body (scale bar: 100 *μ*m). (**e**) HE staining of zebrafish (5 dpf) swim bladders after the LPS challenge (scale bar: 20 *μ*m)

Microinjections of multiple concentrations of LPS, Poly IC or Nano-SiO_2_ into the zebrafish swim bladders were conducted. After the injections, GFP-labeled neutrophils migrated into the swim bladders of the *Tg(mpo:GFP)* zebrafish (Figure 1c, Supplementary Figures S1A and B); neutrophilic accumulation in the swim bladders was time- and dose-dependent. The neutrophil number peaked at approximately 4 hpi (hours post injection) (Supplementary Figures S2A–C). IL8 and MIP-2, which are commonly accepted neutrophil-associated chemokines,^36, 37^ were injected into the swim bladders to assess the efficiency of this *in vivo* model. As expected, neutrophils migrated into the chemokine-injected swim bladders (Supplementary Figures S1C, D, S2D and E).

Taken together, the injected exogenous materials triggered significant neutrophilic infiltrations into the swim bladders.

### Neutrophils are recruited into the LPS-injected swim bladder

In the LPS-induced ALI animal model, neutrophil activation is an essential component of a protective host immune response. However, overzealous activation triggers a tremendous neutrophilic influx into the inflammatory site, thereby resulting in tissue damage.^8^ Investigations of neutrophil recruitment were performed after LPS injections into the swim bladders, including microscopic imaging, pathologic analyses and assessments of neutrophilic migration dynamics in *Tg(mpo:GFP/flk1:mCherry)*^38^ zebrafish with fluorescently labeled vessels and neutrophils.

**Figure 2:**
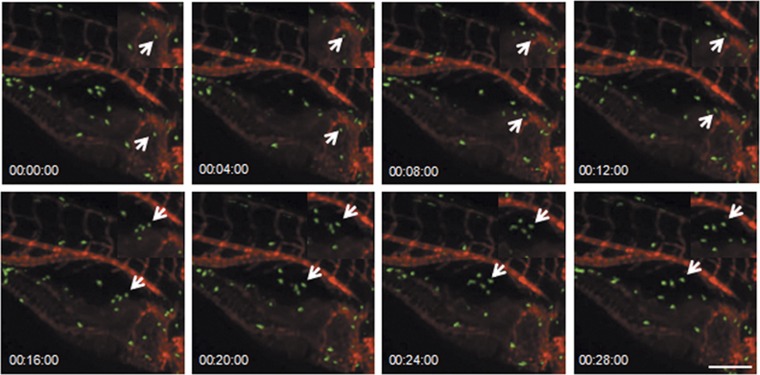
Neutrophils are recruited to the LPS-injected swim bladders *in vivo*. Neutrophils accumulate in the swim bladders of *Tg(mpo:GFP/flk1:mCherry)* zebrafish larva after the LPS (50 ng) injection (green, neutrophil; red, vessels; z-stack: 31 sections every 5 *μ*m; scale bar: 70 *μ*m; arrow indicates a neutrophil that is moving into the swim bladder; time is expressed as h:min:s starting at 40 min post injection; see Supplementary Movie S1). The magnification is presented in the top right corner of each image

An analysis of the neutrophil numbers relative to the stimulant dosages revealed that LPS produced the most significant neutrophil accumulation at 4 hpi compared with Poly IC and Nano-SiO_2_ (Figure 1a). The neutrophil infiltration from the distal end mostly favored the swim bladders over other sites in the zebrafish. This was observed in the global (Figure 1d) and local microscopic (Figure 1b) fields. Consistent with this, a hematoxylin and eosin (HE)-based evaluation showed that the cellular infiltration was increased in the LPS model (Figure 1e). To observe the process of neutrophil recruitment, real-time confocal microscopy was performed in *Tg(mpo:GFP/flk1:mCherry)* zebrafish. Blood capillary and major blood vessels (indicated by red fluorescence) were apparent in the transgenic zebrafish, and an outline of the swim bladder, which was surrounded by blood vessels, was visible. Neutrophils actively moved into the LPS-injected swim bladder (Figure 2 and Supplementary Movie S1). Neutrophils entered the swim bladder through a capillary that surrounded the pneumatic duct or through a site near the pronephric duct. A few neutrophils entered the swim bladder through the adjacent main vessels and migrated away. 3D-reconstruction analysis clearly show that recruited neutrophils aggregated in the lumen of the swim bladder following LPS injection (Supplementary Movies S2 and 3).

In summary, LPS caused an apparent *in vivo* neutrophilic infiltration of the swim bladder in this model.

### LPS induces an inflammatory cytokine response in the whole body and swim bladder of zebrafish

ALI is characterized by inflammatory cytokine expression (i.e., IL-1*β*, IL-6, TNF-*α* and TNF-*β*). To clarify cytokine roles and responses in the LPS-induced swim bladder model, we investigated changes in the mRNA levels of IL-1*β*, IL-6, TNF-*α* and TNF-*β* by quantitative real-time PCR at different time points (0, 1, 2, 4, 8, 12, 18 and 24 hpi).

**Figure 3:**
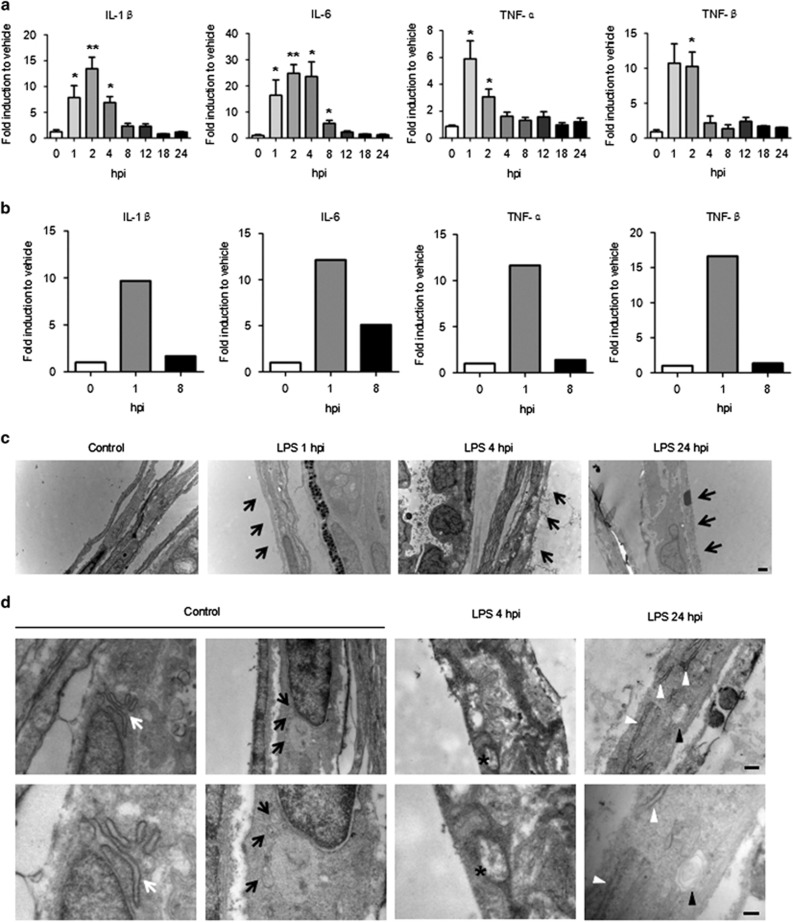
LPS induces inflammatory cytokine expression and injury in the zebrafish swim bladder. (**a**) Whole-body mRNA levels for IL-1*β*, IL-6, TNF-*α* and TNF-*β* increased after the 50-ng LPS injection. The results are presented as the mean±S.E.M.; *n*=30 in each group; *P<0.05; **P<0.01; (Student's *t*-test); three independent experiments. (**b**) The changes to the mRNA levels for IL-1*β*, IL-6, TNF-*α* and TNF-*β* in the swim bladder after the 50-ng LPS injection. *n*=100 swim bladders per group. (**c**) Damaged epithelial layer in the swim bladder of LPS-injected (50 ng) zebrafish larvae at 5 dpf (indicated by black arrows). (**d**) Swollen ER (indicated by white arrowheads) and the control (indicated by white arrows). Mitochondrial injuries include mitochondrial cristae breakage (indicated by asterisks) and vacuolation (indicated by black arrowheads). The control mitochondria are indicated by black arrows. The lowers panels (scale bar: 0.25 *μ*m) are magnifications of the upper panels (scale bar: 0.5 *μ*m)

In the whole-body zebrafish samples, the mRNA levels for IL-1*β* and IL-6 significantly increased at 1 hpi and became higher at 2 hpi compared with 0 hpi. After 8 hpi, their expression levels approached their baselines. The TNF-*α* and TNF-*β* transcripts were promptly transcribed after the LPS challenge, and their levels were elevated within 2 hpi (Figure 3a). Similarly, cytokine induction in the swim bladder after the LPS stimulation was clearly high at 1 hpi relative to 0 and 8 hpi (Figure 3b).

In summary, LPS administration elicited whole body and swim bladder inflammatory cytokine responses in zebrafish.

### Swim bladder injuries are caused by LPS injections

To further define the injuries caused by LPS in the swim bladder, an ultrastructural determination was performed by transmission electron microscopy (TEM). For TEM imaging, partial sections of the swim bladders from control and LPS-treated larvae were generated. The swim bladder's epithelial barrier is the first line of protection from exogenous infection and stimulation of this air-filled cavity.^39^ Distortions of this epithelial lining were observed in the experimental group after the LPS challenge, whereas the control group exhibited a normal epithelial structure. The broken and dispersive epithelium was most apparent at 4 hpi compared with the epithelial damage observed at 1 or 24 hpi (Figure 3c). Additionally, endoplasmic reticulum (ER) swelling with an unsmooth margin was induced by LPS at 24 hpi. Deficient epithelial mitochondria with breakages and vacuolations in the mitochondrial cristae were identified at 4 and 24 hpi, respectively (Figure 3d).

The LPS-injected swim bladder showed identifiable microscopic signs, including injuries to the epithelium, ER and mitochondria. The direct damage to the microstructure of the swim bladder contributed to the effects of the LPS injection.

### Shp2 was identified by its inhibitory effect on neutrophilic infiltration

**Figure 4:**
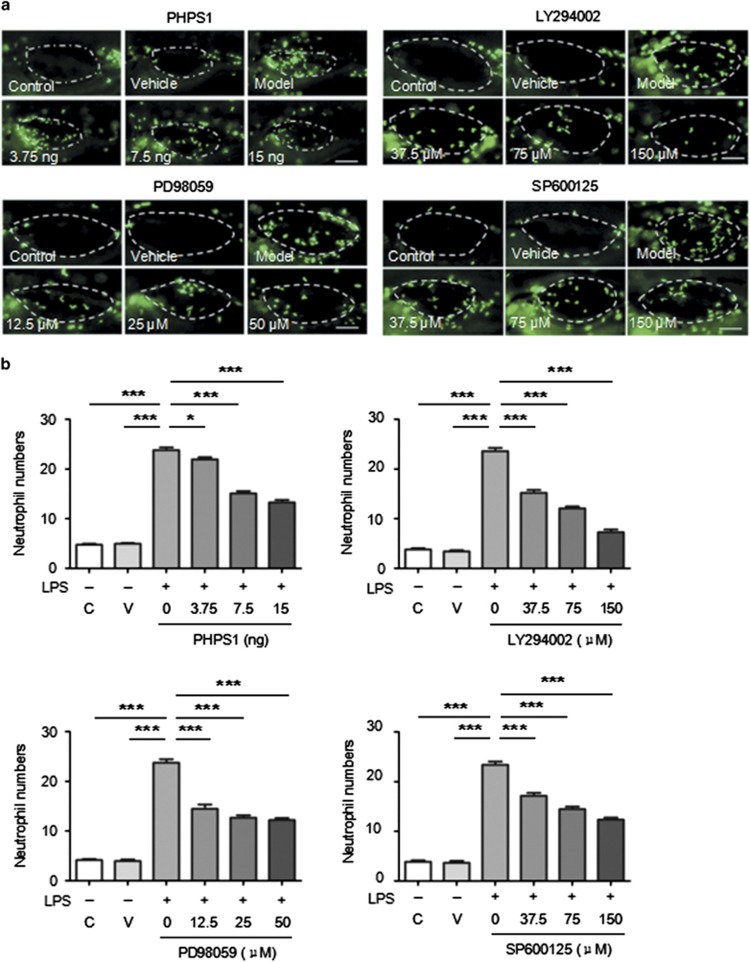
Inhibitors affect neutrophil recruitment into the swim bladder after LPS injection. (**a**) Administration of PHPS1, LY294002, PD98059 and SP600125 inhibited neutrophil recruitment after the LPS injection (dotted line indicates swim bladders; scale bar: 100 *μ*m). (**b**) The corresponding statistical graph (C, control; V, vehicle). The results are presented as the mean±S.E.M.; *n*=15 per group; *P<0.05; ***P<0.001 (Student's *t*-test)

The larval transparency and easy transgenesis of zebrafish have made them increasingly useful for physiological assessments of chemical compounds in vertebrate organisms.^40^ To further identify key genes for neutrophil recruitment into the swim bladder, an inhibitor library of tyrosine kinases and phosphatases was used to screen targets. This approach validated the effectiveness of PI3K, Erk and Jnk in influencing neutrophil migration into the LPS-infected swim bladder. Shp2 (Src homology domain-containing tyrosine phosphatase 2) was also observed in our study as having novel effects on promoting neutrophil migration. LY294002 (a PI3K inhibitor), PD98059 (an Erk inhibitor), SP600125 (a Jnk inhibitor) and PHPS1 (a Shp2 inhibitor) suppressed neutrophil recruitment into the swim bladder (Figures 4a and b). Therapeutic effects were examined by delivering these inhibitors 2 h after the LPS injection. The results showed that LY294002, PD98059, SP600125 and PHPS1 inhibited neutrophil recruitment after ALI was established in the swim bladder (Supplementary Figures S4A and B). The inhibitory effect of PHPS1 on neutrophil migration into the swim bladder suggests that Shp2, a previously described contributor to ALI-associated endothelial disruption and edema formation,^41^ is a potential therapeutic target for ALI.

### Shp2 deficiency alleviates LPS-induced inflammation in an ALI mouse model

**Figure 5:**
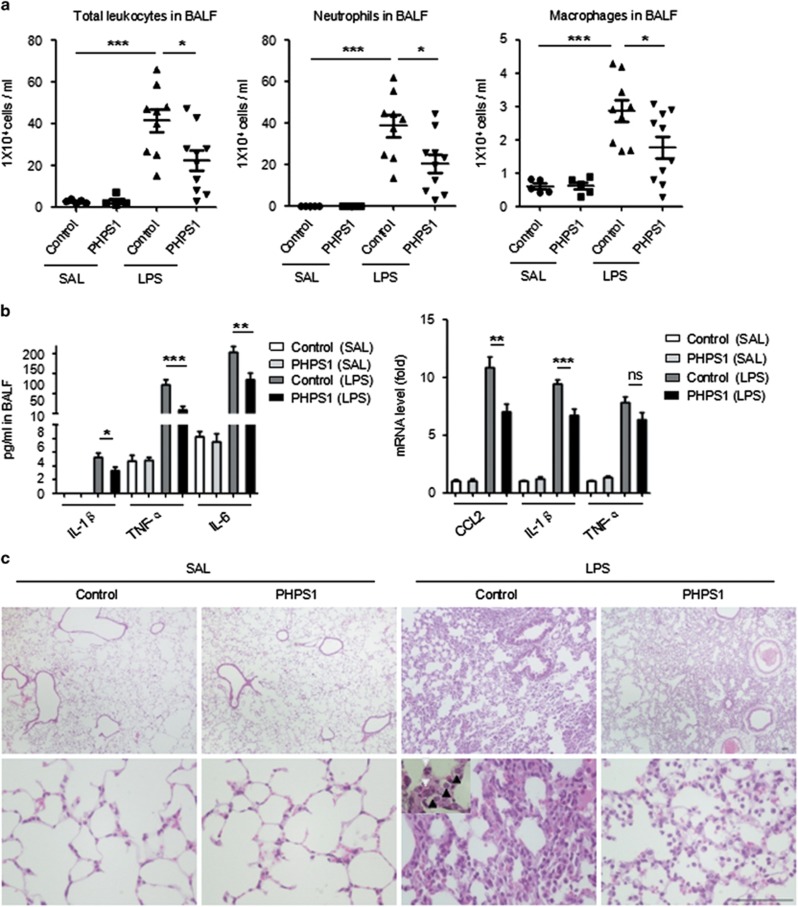
Shp2 inhibition relieves LPS-induced inflammation in the murine lung. (**a**) BALF analysis of control (Shp2^fl/fl^) and PHPS1-treated mice that were challenged with saline or LPS (3 mg/kg) for 24 h. The number of total leukocytes, neutrophils and macrophages decreased in BALF after Shp2 inhibition (*n*=10) or knockout (*n*=8). The results are presented as the mean±S.E.M.; *P<0.05; **P<0.01; ***P<0.001 (Student's *t*-test). (**b**) The IL-1*β*, IL-6, and TNF-*α* levels in the BALF and the CCL2, IL-1*β* and TNF-*α* mRNA levels in the lung decreased in the PHPS1-treated mice relative to the controls. The results are presented as the mean±S.E.M.; *P<0.05; **P<0.01; ***P<0.001; NS, not significant (Student's *t*-test). Three independent experiments. (**c**) HE stains of lung sections 24 h after the saline or LPS challenge (upper panels, scale bar: 100 *μ*m; lower panels, scale bar: 100 *μ*m). White arrowheads indicate neutrophils; black arrowheads indicate macrophages

**Figure 6:**
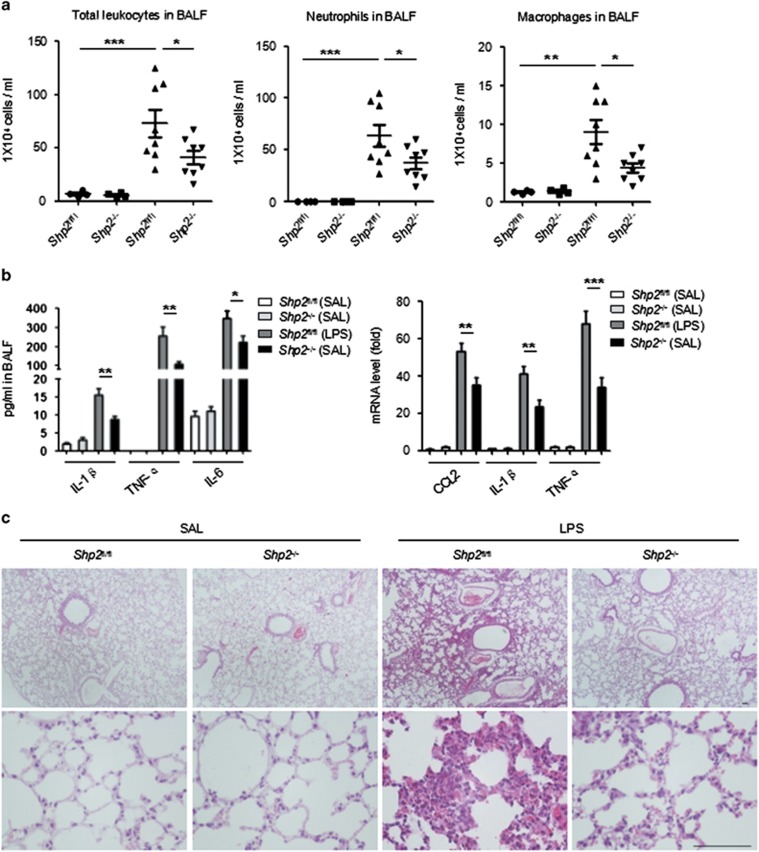
Shp2 deficiency relieves LPS-induced inflammation in the murine lung. (**a**) BALF analysis of control (Shp2^fl/fl^) and Shp2^−/−^ mice that were challenged with saline or LPS (3 mg/kg) for 24 h. The number of total leukocytes, neutrophils and macrophages decreased in the BALF after Shp2 inhibition (*n*=10) or knockout (*n*=8). The results are presented as the mean±S.E.M.; *P<0.05; **P<0.01; ***P<0.001 (Student's *t*-test). (**b**) The IL-1*β*, IL-6 and TNF-*α* levels in the BALF and the CCL2, IL-1*β* and TNF-*α* mRNA levels in the lung decreased in the Shp2^−/−^ mice relative to the controls. The results are presented as the mean±S.E.M.; *P<0.05; **P<0.01; ***P<0.001; NS, not significant (Student's *t*-test). Three independent experiments. (**c**) HE stains of lung sections 24 h after the saline or LPS challenge (upper panels, scale bar: 100 *μ*m; lower panels, scale bar: 100 *μ*m)

To verify the influence of Shp2 on neutrophil infiltration in LPS-induced ALI, we administered the Shp2 inhibitor PHPS1 to mice by peritoneal injection. PHPS1 significantly reduced the pulmonary infiltration of neutrophils, macrophages and total leukocytes in LPS-induced ALI mice (Figure 5a). PHPS1 also lessened lung inflammation, which was characterized by reduced protein levels of pro-inflammatory cytokines in BALF (TNF-*α*, IL-6) and decreased mRNA expression in the lung tissues (CCL2, IL-1*β*) (Figure 5b). A histological examination of the alveolar structure indicated that the lung destruction and injuries in the ALI mice were alleviated by the PHPS1 treatment, compared with the vehicle (DMSO)-treated group (Figure 5c). The myeloid cell-specific LysM-Cre line^42^ was used to inactivate Shp2 to further investigate its function in pulmonary neutrophil recruitment. In agreement with the result in the PHPS1-treated mice, the Shp2^−/−^ mice in the ALI model were characterized by decreased pulmonary leukocyte infiltration (Figure 6a), lower levels of pro-inflammatory cytokines in the lungs (Figure 6b) and milder inflammation-associated alveolar damage (Figure 6c) compared with their littermates.

As a potential evaluation model for lung injury, the swim bladder model was applied to assess the severity of pneumonia. The BALF samples were collected from patients suffering from pneumonia and tracheal foreign bodies. The pneumonic BALF that was microinjected into the swim bladders induced significant neutrophil recruitment compared with the control BALF from the tracheal foreign body group (Supplementary Figure S3).

The loss of Shp2 inhibited their migration to the LPS-induced inflammatory site, which partially protected mice from ALI. These data support our prediction of a regulatory role for Shp2 in pulmonary neutrophilic infiltration during ALI. Moreover, this swim bladder model is a promising new model for the effective evaluation of lung injuries.

## Discussion

In this article, we describe a novel and useful zebrafish model for investigating neutrophilic infiltration during ALI. This swim bladder model was successfully established to detect real-time neutrophil recruitment into the inflammatory site. We used this transparent *in vivo* system to dissect the neutrophil response after a swim bladder infection. Using this relatively high-throughput system, we became the first to identify Shp2 as a participant in neutrophil migration during ALI. Moreover, pulmonary inflammation in ALI mice was alleviated by Shp2 inhibition and disruption. This swim bladder model provides unique opportunities for exploring the mechanisms that underlie ALI and potential therapeutic targets.

The alveolar microenvironment is vulnerable to exogenous stimulation by bacterial infections, viral infections and air pollutants. Severe injuries in the alveolar microenvironment, including epithelial and endothelial injuries, microvascular thrombi and pulmonary edema, can lead to ALI/ARDS.^12^ *Streptococcus pneumonia-*induced alveolar infections can increase alveolar permeability and leukocyte accumulation and decrease surfactant protein D.^43^ The 2009 H1N1 pandemic influenza infection also increased neutrophil levels throughout the alveolar space and enhanced inflammation and immune cell infiltration into the lung.^44^ Investigations of host–pathogen interactions in the alveolar space will contribute to our understanding of lung diseases such as ALI/ARDS. However, the pulmonary alveoli that are located at the terminal ends of the distal airways are inaccessible to *in vivo* functional approaches. Because the zebrafish swim bladder and lung exhibit multiple similarities, we utilized the swim bladder as an *in vivo* model for the alveolar space. The alveolar injuries caused by bacteria, viruses and inhaled air pollutants were modeled by injecting exogenous materials (LPS, Poly IC, Nano-SiO_2_) into swim bladders. Using this transparent system, significant neutrophil aggregation was detected in *Tg(mpo:GFP)* zebrafish after infection. *In situ* injuries that exhibited epithelial disruptions, ER swelling and mitochondrial deficiencies occurred after LPS injections into the swim bladders. The swim bladder injuries were characterized by the elevated expression levels of inflammatory cytokines, such as IL-1*β*, IL-6, TNF-*α* and TNF-*β*. Importantly, the pathological characteristics of ALI-associated alveolar neutrophilic infiltration were partially reproduced in this system. This indicates that the swim bladder can be used as an effective tool for dissecting the mechanisms of alveolar neutrophilic infiltration during ALI.

Because the ALI-associated increases in permeability and edema formation are neutrophil-dependent, alveolar neutrophilic infiltration is regarded as a contributor to ALI progression.^8^ The mechanisms of neutrophil chemotaxis have been previously dissected to decipher ALI pathogenesis.^9, 45^ Here, our swim bladder model offers an *in vivo* system for the direct observation of real-time neutrophil dynamics during ALI. This system will accelerate future investigations of neutrophilic recruitment during ALI.

Different animal models for ALI, including mouse, rat and rabbit, have been previously reported.^12^ For modeling and investigating neutrophilic alveolitis in ALI, this zebrafish swim bladder model has advantages in dynamic neutrophil imaging and genetic manipulation over other animal models. However, none of the animal models can adequately reproduce every feature of human ALI. Species differences between human and animal models, which may involve Toll-like receptors, chemokines or chemokine receptors, mononuclear phagocyte system and nitric oxide,^12^ limit the applications of this swim bladder model. Nonetheless, its intrinsic advantages make the zebrafish swim bladder model uniquely capable of promoting ALI investigations and treatments.

Zebrafish are increasingly used in large-scale genetic and therapeutic screening projects.^13^ Using the swim bladder model, Shp2 was associated with neutrophilic alveolitis in ALI. PHPS1 administration and Shp2 disruption in myeloid cells successfully reduced inflammation in LPS-induced ALI mice. For the first time, Shp2 has been shown to participate in neutrophil recruitment in ALI. Therefore, this model can be utilized as a drug and genetic screening tool for ALI and other lung diseases.

The mouse model used in this study contains Shp2 deletions in monocyte/macrophages. It is possible that the inhibitors function indirectly by inhibiting chemoattractant production. Neutrophil activation and recruitment are regarded as key components of ALI/ARDS progression, so we focused our study on neutrophil accumulation without addressing macrophages and other cell types. In the myeloid KO mice, the functional inhibition of other cell types cannot be ignored, and future investigations should evaluate these further, along with the specific roles of the various cell types.

Although there have been numerous advances in modeling and evaluating lung diseases (i.e., 3D cell culture models^46^), suitable animal models for *in vivo* analyses remain sparse.^47^ Our preliminary data show that the BALF samples from pneumonic patients significantly triggered neutrophil aggregation in the swim bladder compared with the control. However, further investigations of the BALF-induced neutrophil response are necessary. Moreover, the swim bladder model can be used as a potential evaluation model for assessing the severity of pneumonia and other lung diseases.

In summary, this relatively high-throughput model provides new insights into the etiology of neutrophilic alveolitis in ALI and promotes the discovery and development of new molecular therapeutic targets for this currently refractory disease. Moreover, this *in vivo* system is a promising and effective evaluation model for lung injuries that will improve the modeling of pulmonary disorders.

## Materials and Methods

### Zebrafish care and maintenance

Zebrafish were housed in a light- and temperature-controlled aquaculture facility at Hunter Biotechnology, Inc., under a standard 14/10 h light/dark photoperiod. The water temperature was maintained at 28 °C. The fish were fed live brine shrimp twice daily. Four to five pairs of adult zebrafish were set up for natural mating, and 200–300 embryos were typically generated. Embryos and larvae were maintained at 28 °C in fish water (0.2% Instant Ocean Salt in deionized water, pH 6.9–7.2, conductivity 480–510 *μ*S/cm and hardness 53.7–71.6 mg per  l CaCO_3_). For experiments, the embryos were collected, washed and chosen at 6 and 24  h.p.f. (hours post fertilization). The zebrafish facility at Hunter Biotechnology, Inc. (Hangzhou, China) is accredited by the Association for Assessment and Accreditation of Laboratory Animal Care (AAALAC) International. This study's fish lines included *Tg(mpo:GFP)*^34^ and *Tg(mpo:GFP/flk1:mCherry)* (*Tg(mpo:GFP)* crossed with *Tg(flk1:mCherry)*^38^). The transgenic *Tg(mpo:GFP)* zebrafish contained GFP-expressing neutrophils; *Tg(mpo:GFP/flk1:mCherry)* exhibited neutrophil-specific GFP expression and vessel-specific red fluorescent protein (mCherry) expression.

### Swim bladder injections

At 5 dpf, larvae with inflated swim bladders were selected for microinjection. After anesthetization (0.03% Tricaine (Sigma, St Louis, MO, USA)), zebrafish were loaded on a customized microplate that was specifically designed for microinjection. The fish were microinjected with different doses of LPS (Sigma), Poly IC (Sigma), Nano-SiO_2_ (Aladdin, Shanghai, China), IL8 (Peprotech, Rocky Hill, NJ, USA) and MIP-2 (Peprotech) in 5 nl of PBS, and an equal volume of PBS was injected as the control. The designated concentration of each chemical was loaded into a pulled glass capillary needle (World Precision Instruments, Sarasota, FL, USA) made by an electrode puller (Narishige, PC-10, Tokyo, Japan). The needles were trimmed to internal diameters of 15 *μ*m and outer diameters of 18 *μ*m. The microneedle was attached to a Microinjector (Zgenebio, PCO-1500, Taipei, Taiwan) to perform swim bladder injections as previously described.^35^ Larvae with the correct inoculum location were selected and used for further experiments.

### Inhibitor treatment

At 5 dpf, zebrafish were anesthetized and loaded onto the microplate as described above. For the inhibitor screening assay, indicated inhibitors from the kinase and phosphatase bank (Merck Millipore, Darmstadt, Germany) were delivered by soaking or microinjection. PHPS1 (dissolved in 10% DMSO) was delivered by intravenous microinjection.^48^ The LY294002, PD98059 and SP600125 stock solutions were prepared with 100% DMSO and delivered to zebrafish by soaking. The inhibitor treatment results were collected at 4 hpi. Specific dose information is presented in the relevant figure legends

### Human BALF injections

BALF was collected from patients suffering from pneumonia or tracheal foreign bodies. The BALF samples were microinjected into the swim bladders of *Tg(mpo:GFP)* zebrafish to detect neutrophil migration. The protein levels in BALF were measured with the Enhanced BCA Protein Assay Kit (Beyotime, Beijing, China).

### Fluorescence microscopy

For live imaging, zebrafish were anesthetized with 0.03% Tricaine (Sigma Aldrich, St Louis, MO, USA) and immobilized with 1% LMA (low melting point agarose) on a 35-mm glass-bottom Petri dish (Nest, Rahway, NJ, USA). Confocal microscopy was performed using an Olympus IX-81 (Olympus, Tokyo, Japan) inverted microscope with a Yokogawa (Yokogawa, Tokyo, Japan) CSU-X1 spinning disk and a 10 × /0.4 NA objective lens. GFP and mCherry were detected by excitation at 488 and 561 nm, respectively. Images were collected, and Z-series images were stacked or 3D-reconstructed with ImageJ or MetaMorph. Time-lapse spinning disk confocal microscopy was performed over 6 h. Z-stack images were obtained every 2 min, and movies were generated with MetaMorph. The imaging process was performed on a heated stage at 28.5 °C with a 5-*μ*m step size. The time intervals and total time periods are specified in the relevant figure legends. The maximal projections of fluorescent image channels are overlaid with a single slice of the differential interference contrast channel or are independently presented. The number of slices for each maximum projection is indicated in the relevant figure legends. For time–dose imaging, pictures were acquired with a multi-purpose zoom fluorescence microscope (AZ100; Nikon, Tokyo, Japan) using a × 2/0.2 NA objective lens.

### TEM

For TEM imaging, larvae were maintained for 5 days and killed by Tricaine overdose. Samples were fixed with 2.5% glutaraldehyde in phosphate buffer (0.1 M, pH 7.0) and incubated in the fixative for at least 24 h. Each fish was rinsed with phosphate buffer (0.1 M, pH 7.0) three times for 15 min, postfixed with 1% osmium tetroxide for 90 min and washed three times in phosphate buffer (0.1 M, pH 7.0) for 15 min after each step. Each specimen was dehydrated using increasing concentrations of ethanol (30, 50, 70, 80, 90, 95 and 100%) for 15 min per step and transferred to absolute acetone for 20 min. The samples were placed in a 1:1 mixture of absolute acetone and resin for 1 h at room temperature and transferred to a 1:3 mixture of absolute acetone and resin for 3 h. Samples were subsequently embedded in an epoxy resin medium overnight. The specimens were placed in resin-containing Eppendorf tubes and heated at 70 °C for 9 h or more. The specimens were sectioned using a LEICA EM UC7 ultratome, and 1-*μ*m-thick sections were stained with toluidine blue for orientation and light microscopy. Ultrathin 70-nm-thick sections were cut with a diamond knife and stained with uranyl acetate and alkaline lead citrate for 5 and 10 min, respectively. The samples were examined with a Hitachi Model H-7650 TEM using 80 kV. The magnification times are indicated in each figure.

### Histopathology

The 5-dpf zebrafish and the left lung tissues from the experimental mice were fixed in formalin and embedded in paraffin. For the histological analysis, the paraffin-containing samples were sectioned and stained with hematoxylin and eosin according to the standard procedure. The HE-stained slides were examined by optical microscopy (Eclipse Ci-S; Nikon).

### RNA isolation and quantitative real-time PCR

Total RNA from 30 whole larvae, 100 dissected swim bladders and the murine lung tissues were extracted using Trizol (Invitrogen, Carlsbad, CA, USA) according to the manufacturer's instructions. The RNA concentration was measured using a Nanodrop2000 (Thermo Fisher, Lafayette, CO, USA). Total RNA was reverse transcribed using the ReverTraAce quantitative PCR (qPCR) RT kit (Toyobo, Osaka, Japan), and real-time qPCR was performed using the UltraSYBR Mixture (Cwbio, Beijing, China). The qPCR primer sequences used in this study are shown in Table 1. The qPCR analysis was performed with a LightCycler480 (LC480II; Roche, Basel, Switzerland) using the following conditions: 10 min at 95 °C, followed by 40 cycles of 15 s at 95 °C and 1 min at 60 °C, and a final dissociation between 65 and 95 °C. Threshold cycles (Ct) and dissociation curves were analyzed with the LightCycler480 (Roche). Gene expression levels were normalized to actin (ΔCt) and compared with the control (ΔΔCt). Fold induction (2^ΔΔCt^) is presented in this report.

### Mice and ALI model

C57BL/6 *Shp2*^−/−^ (*LysM*^cre/+^:*Shp2*^flox/flox^) mice and their WT littermates (*LysM*^+/+^:*Shp2*^flox/flox^) were used in this study and have been previously described.^42^ To investigate the effects of PHPS1 on lung inflammation and remodeling, C57BL/6 mice were treated with 3 mg/kg PHPS1 (dissolved in saline with 0.5% DMSO) by intraperitoneal injection before LPS administration. As controls, the C57BL/6 littermates were injected with equal volumes of saline with 0.5% DMSO and treated with LPS. Shp2^−^^/−^ mice were challenged with LPS in sterile saline by airway injection, with Shp2^fl/fl^ mice used as controls. The LPS concentration was 3 mg per kg body weight. After 24 h, the mice were killed for pathological examinations. All mice were 6–8 weeks old.

### Murine BALF analysis and ELISAN

The mice were killed 24 h after the LPS challenge for the BALF analysis and ELISA. With the left bronchus tied, each mouse was lavaged with 0.5 ml of calcium- and magnesium-free PBS into the right lung. This procedure was repeated three times (total approximate volume was 1.5 ml, recovery >80%). BALF from each mouse was collected in an Eppendorf tube, cooled on ice and centrifuged at 1000 r.p.m. at 4 °C for 30 min. Cell pellets were resuspended in 100 *μ*l of PBS. The total number of leukocytes was counted with a Neubauer chamber (Qiujing, Shanghai, China). Differential cell counting was performed by Wright-Giemsa staining according to standard morphological criteria. The BALF supernatant from each experimental mouse was stored at −80 °C until the cytokine measurements. ELISA kits (IL-1*β*, TNF-*α* and IL-6; eBioscience, San Diego, CA, USA) were used for the cytokine analysis. Each sample was measured in accordance with the manufacturer's recommendations.

### Data analysis and statistics

Data analyses were performed with a one-way ANOVA or a two-tailed Student's *t-*test using GraphPad Prism version 5.0 (GraphPad, La Jolla, CA, USA). The number of experimental repeats and larvae are indicated in the relevant figure legends. A *P*-value <0.05 was considered significant and is indicated in the figures as *(*P*<0.05), **(*P*<0.01) or ***(*P*<0.001).

### Ethics statement

All animal protocols and experiments for this study were approved by the Institutional Animal Care and Use Committee of Zhejiang University School of Medicine.

## Figures and Tables

**Table 1:** Genes and primers used in qPCR

Gene	*Sequence (5′ –3′)*	*Reference*
*Zebrafish*		
*β*-actin	Fwd: ATGGATGATGAAATTGCCGCAC	^49^
	Rev: ACCATCACCAGAGTCCATCACG	
IL-1*β*	Fwd: TTGAAAGTGCGCTTCAGCA	^50^
	Rev: CGGTCTCCTTCCTGAAGAACA	
IL-6	Fwd: AGACCGCTGCCTGTCTAAAA	^51^
	Rev: TTTGATGTCGTTCACCAGGA	
TNF-*α*	Fwd: GGCTGGAAAACAACGAGATCA	^50^
	Rev: AAGATCAAAGACGGCTCCAA	
TNF-*β*	Fwd: TCAGAAACCCAACAGAGAACATC	^24^
	Rev: ACCCATTTCAGCGATTGTCC	
		
*Mouse*		
*β*-actin	Fwd: GTGACGTTGACATCCGTAAAGA	This study
	Rev: GCCGGACTCATCGTACTCC	
CCL2	Fwd: TAAAAACCTGGATCGGAACCAAA	
	Rev: GCATTAGCTTCAGATTTACGGGT	
IL-1*β*	Fwd: GAAATGCCACCTTTTGACAGTG	
	Rev: TGGATGCTCTCATCAGGACAG	
TNF-*α*	Fwd: CAGGCGGTGCCTATGTCTC	
	Rev: CGATCACCCCGAAGTTCAGTAG	

## Abbreviations

ALI: acute lung injury
ARDS: acute respiratory distress syndrome
LPS: lipopolysaccharide
Nano-SiO2: silica nanoparticles
ER: endoplasmic reticulum
BALF: bronchoalveolar lavage fluid
GFP: green fluorescent protein
Dpf: days post fertilization
Hpi: hours post injection
HE: hematoxylin and eosin
TEM: transmission electron microscopy
Shp2: Src homology domain-containing tyrosine phosphatase 2
h.p.f.: hours post fertilization
LMA: low melting point agarose
