# Supplementary material for: Manipulating the air-filled zebrafish swim bladder as a neutrophilic inflammation model for acute lung injury
Source: Cell Death Dis. 2016 Nov 10;7(11):e2470–. doi: 10.1038/cddis.2016.365 (PMC5260887; doi:10.1038/cddis.2016.365)
Supplement: Supplementary Information [file cddis2016365x1.doc]

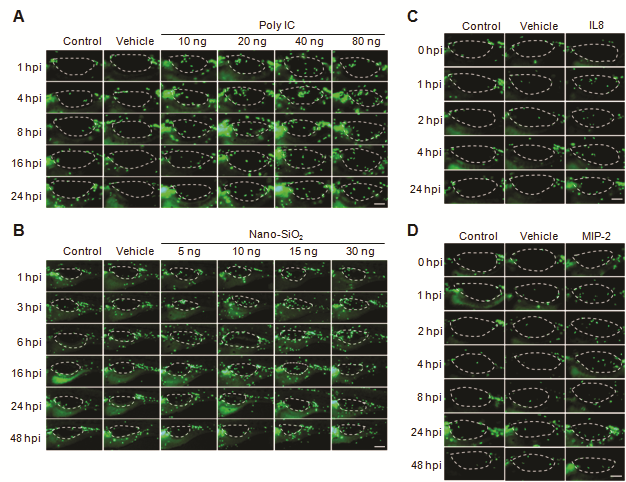


**Figure S1.** **Exogenous stimuli trigger neutrophil recruitment into zebrafish swim bladders.** Neutrophil recruitment to exogenous materials [Poly IC (A), Nano-SiO_2_ (B), IL8 (1 ng) (C), MIP-2 (2 ng) (D)] that were injected into the swim bladders (indicated by dotted line) of *Tg(mpo:GFP)* zebrafish larvae at 5 dpf was monitored (green, neutrophil; scale bar: 100 μm).


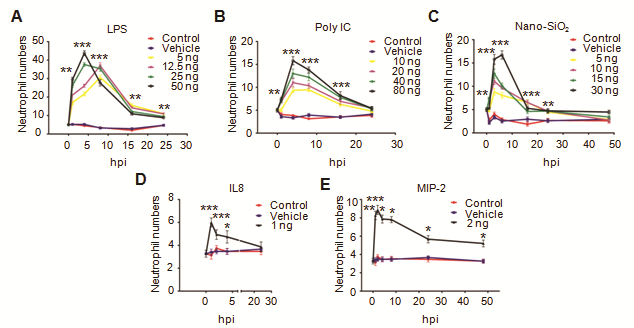


**Figure S2. Statistical diagrams of neutrophil accumulation in the swim bladder after exogenous material injections.** The swim bladders of *Tg(mpo:GFP)* zebrafish larvae were injected at 5 dpf with LPS (A), Poly IC (B), Nano-SiO_2_ (C), IL8 (D), and MIP-2 (E). Neutrophil recruitment to the injection site was examined. The results are presented as the mean±s.e.m.; *n*=15 per group; ******P*<0.05; *******P*<0.01; ********P*<0.001 (Student’s *t*-test).


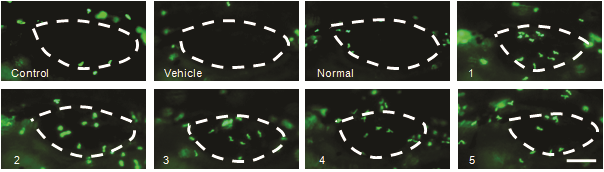


**Figure S3. Injection of BALF from pneumonia patients induces neutrophil migration to the swim bladder.** The swim bladders (indicated by dotted line) of *Tg(mpo:GFP)* zebrafish larvae were injected at 5 dpf with pneumonic BALF (1,2,3,4,5) or BALF from patients suffering from tracheal foreign bodies (Normal). Neutrophil recruitment to the injection site was examined (green, neutrophil; scale bar: 100 μm).


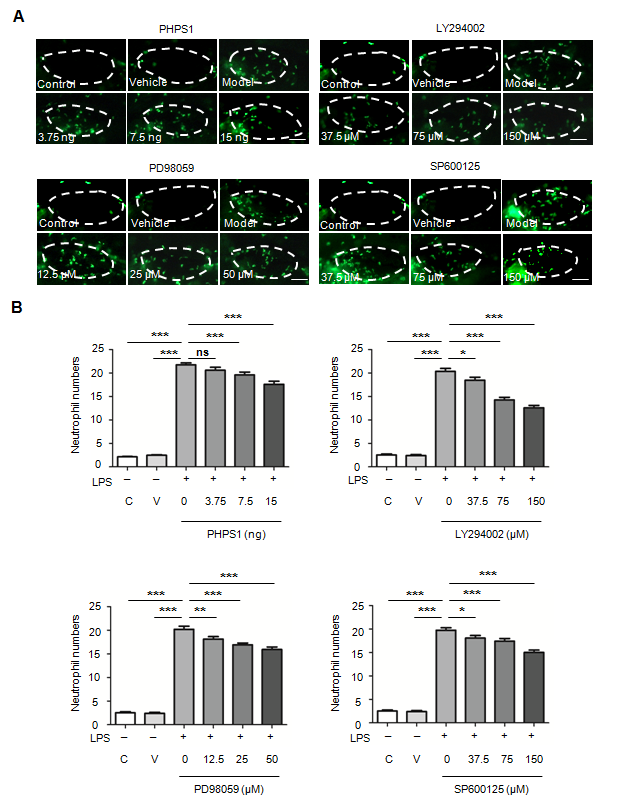


**Figure S4. Inhibitors affect neutrophil recruitment into the swim bladder after ALI is established.** (A) PHPS1, LY294002, PD98059 and SP600125 inhibited neutrophil recruitment after LPS injection (dotted line indicates the swim bladder; scale bar: 100 μm). (B) The corresponding statistical graph (C, control; V, vehicle). The results are presented as the mean±s.e.m.; *n*=15 per group; ******P*<0.05; *******P*<0.01; ********P*<0.001 (Student’s *t*-test).

**Movie 1: Neutrophils migrate to the LPS-injected swim bladder.** Neutrophils show directed movement toward the LPS-injected (50 ng) swim bladders of *Tg(mpo:GFP/flk1:mCherry)* larvae (191 frames every 2 minutes; z-stack: 31 sections every 5 μm; scale bar 70 μm).

**Movie 2: The neutrophil location in the control swim bladder.** Neutrophils did not appear in the control swim bladders of *Tg(mpo:GFP)* larvae (z-stack: 31 sections every 5 μm; scale bar 100 μm).

**Movie 3: The neutrophil location in the LPS-injected swim bladder.** Neutrophils localized to the lumina of the LPS-injected (50 ng) swim bladders of *Tg(mpo:GFP)* larvae (z-stack: 31 sections every 5 μm; scale bar 100 μm).
